# Rare Does Not Mean Worthless: How Rare Diseases Have Shaped Neurodevelopment Research in the NGS Era

**DOI:** 10.3390/biom11111713

**Published:** 2021-11-17

**Authors:** Mattia Zaghi, Federica Banfi, Edoardo Bellini, Alessandro Sessa

**Affiliations:** 1 Stem Cell and Neurogenesis Unit, Division of Neuroscience, IRCCS San Raffaele Scientific Institute, 20132 Milan, Italy; mattia.zaghi@hsr.it (M.Z.); banfi.federica@hsr.it (F.B.); bellini.edoardo@hsr.it (E.B.); 2 CNR Institute of Neuroscience, 20129 Milan, Italy

**Keywords:** next-generation sequencing (NGS), neurodevelopmental disorders (NDDs), diagnosis, experimental modelling, gene function

## Abstract

The advent of next-generation sequencing (NGS) is heavily changing both the diagnosis of human conditions and basic biological research. It is now possible to dig deep inside the genome of hundreds of thousands or even millions of people and find both common and rare genomic variants and to perform detailed phenotypic characterizations of both physiological organs and experimental models. Recent years have seen the introduction of multiple techniques using NGS to profile transcription, DNA and chromatin modifications, protein binding, etc., that are now allowing us to profile cells in bulk or even at a single-cell level. Although rare and ultra-rare diseases only affect a few people, each of these diseases represent scholarly cases from which a great deal can be learned about the pathological and physiological function of genes, pathways, and mechanisms. Therefore, for rare diseases, state-of-the-art investigations using NGS have double valence: their genomic cause (new variants) and the characterize the underlining the mechanisms associated with them (discovery of gene function) can be found. In a non-exhaustive manner, this review will outline the main usage of NGS-based techniques for the diagnosis and characterization of neurodevelopmental disorders (NDDs), under whose umbrella many rare and ultra-rare diseases fall.

## 1. Introduction

The term rare disease is commonly used in the biomedical field and comprises a wide range of conditions that affect different regions of the body. The relevant parameter that should be considered when determining whether a disease can be defined is rare is the population prevalence. Moreover, depending on the country, the population prevalence can be slightly different; hence, in the USA, less than 200 thousand people must be affected, whereas in the EU, the population prevalence refers to 1 in 2000.

Research on diseases that only affect a few thousand, hundred, or even dozens of individuals has always been received with mixed feelings by lay people as well as by (part of) the scientific community. On the one hand, funding agencies and patient advocacy have put a great deal of effort into promoting and financing these investigations, keeping patients and families in direct contact with researchers. On the other hand, public opinion and the pharmaceutical industry lack an understanding of the relevance and impact that studies into these rare diseases might have.

Neurodevelopmental disorders (NDDs) [1] refer to any condition that, for the large majority, represent the archetypal cases of rare diseases. When all of these diseases are considered together, then slightly under 3% of the younger population is affected worldwide [2]. The prototypical features of affected patients are an impaired ability to communicate, behavioral alterations, and speech difficulties that are often connected to intellectual and motor impairments. Although common clinical features are present, the etiology is very heterogeneous; thus, understanding the actual pathogenic mechanism that is attributed to each patient is extremely challenging [1].

The advent of next-generation sequencing (NGS) has revolutionized not only the diagnosis of NDDs but also the research being conducted in this field. Its impact has been remarkable, spanning from understanding the genetic causes of several neglected diseases, leading, for example, to the discovery of new gene associations with complex alterations, such as those associated with autism spectrum disorders (ASDs) or intellectual disabilities (IDs).

This has fostered an interesting aspect of NDD research: in several cases, investigating disease etiology and pathological mechanisms paves the way towards our understanding of the normal development of the nervous system and its physiological functions as well as the discovery of gene functions. Thus, the coupling between the scientific interest in rare diseases and the rising tide of omics techniques has been responsible for the achievement of several milestones for the biomedical community.

Investigating NDDs have allowed for the discovery of many genes that are involved in essential pathways for neural development, such as neural migration, folded structure acquisition (gyrification), and increased brain dimensions. Some examples are the *MCPH* locus, which has been discovered to be strongly associated with microcephaly [3], information that has helped us to unravel the mechanisms that underline human brain expansion; the *LIS1* [4] and *DCX* [5] genes that are associated with the features of NDDs have helped us to unfold the key processes that are essential for brain maturation.

The advent of NGS has significantly enhanced the possibility of researchers to find, study, and understand causal gene variants in this context. In this review, we summarize diverse aspects regarding how NGS has reshaped research on neural development disorders.

## 2. NGS Impact on NDD Diagnosis

### 2.1. Whole Exome Sequencing and the Discoveries of New Variants

Before NGS, time- and money-consuming processes were necessary to obtain sufficient data quality and statistical significance to discover new genetic variants, either de novo or familial, that are involved in disease pathogenesis.

The first decade of the new millennium (the 2010s) marked a significant change in the diagnosis of NDDs. The massive deployment of NGS has allowed the discovery of several genes that were not previously described as being disease causing. Whole exome sequencing (WES) was the main driver of this revolution in the early days, and it is a technique that specifically enriches the protein-coding DNA portions of the genome. These portions were later sequenced at a very high coverage rate, allowing the statistical significance to call them variants of clinical interest [6,7]. Despite this, these DNA portions account for just 1% of the total genome, which contains all of the genetic information encoding the actual proteins, making it very enticing to sequence the entire at an affordable cost, allowing us to obtain highly informative DNA content.

Some examples of new disease-associated genes that have been discovered using NGS technologies are DHODH [8], the causative gene of the Miller syndrome, WDR35, the gene that is responsible for Sensenbrenner syndrome [9], and SETBP1, which is responsible for the Schinzel–Giedion syndrome (SGS) [10]. Strikingly, the use of NGS has enabled scientists to solve an endless puzzle that has remained unsolvable for years. Indeed, the first SGS patient was reported in 1978 [11], the first patient affected by Sensenbrenner syndrome was reported in 1975 [12], and the first Miller syndrome patient was reported in 1979 [13]. Despite these syndromes being described long ago, the complexity of the symptoms and the rarity of affected individuals made it impossible to discover the causative genes for these diseases with the technologies that were available in the pre-NGS era.

In subsequent year, an extensive study involving several patients affected by ASDs, again exploiting WES technology, led to the discovery of the *FOXP1*, *GRIN2B*, *SCN1A,* and *LAMC3* gene variants being involved in the pathogenesis of sporadic forms [14]. Other studies that had made use of this same approach on a larger cohort of patients found that ASD-associated variants are significantly enriched with missense mutations and splice site alterations [15].

Within this framework, studies that are sustained by a large consortium and foundations have helped expand the efforts to discover new variants. A concrete example is the Simons Foundation Autism Research Initiative (SFARI), which was founded in 2006. SFARI has built one of the biggest databases containing ASD-associated genes and remains as one of the biggest resources in the field of autism research. It contains 1089 genes that are the results of 2912 associated variants, the majority of which (2462) are rare variants. In fact, all of the variants that are present in this database were manually annotated form peer-reviewed articles.

Another example is the Autism Sequencing Consortium, which was founded in 2010 by Dr. Joseph D. Buxbaum in order to foster collaboration and data sharing among scientists in the field. Additionally, the UK10K Consortium, which was designed for a broader scope, has also provided a huge contribution in the field of genetic association with NDDs. The aim of this project is to fully sequence the genome of thousands of individuals and to monitor their health through their lifetime at the same time to possibly understand genetic contributions to different diseases and their interaction with other environmental factors.

Considering that these big consortiums were created based upon data obtained using NGS, we can easily understand how NGS is considered to be a game changer for biomedical research.

Thanks to this and other efforts that have been made to finance research into variant discovery, many large studies that include more patients have helped strong associations be found.

### 2.2. Sequencing Contribution in Guiding Biological Questions

The extensive use of NGS has not only helped from a purely diagnostic standpoint. Parallel to the discovery of new variants and associated genes, NGS has helped researchers better understand the biological pathways that are involved in the pathogenesis of NDDs. Large studies that have taken advantage of WES techniques were at the forefront of this new trend, revealing important concepts regarding the type of mutations and the pathways involved in the pathogenesis of ASDs [16,17]. More specifically, the studies using this technology have disclosed how most ASD-associated mutations are loss of function (LoF) mutations, with many of them being clustered in specific genes.

**Figure 1:**
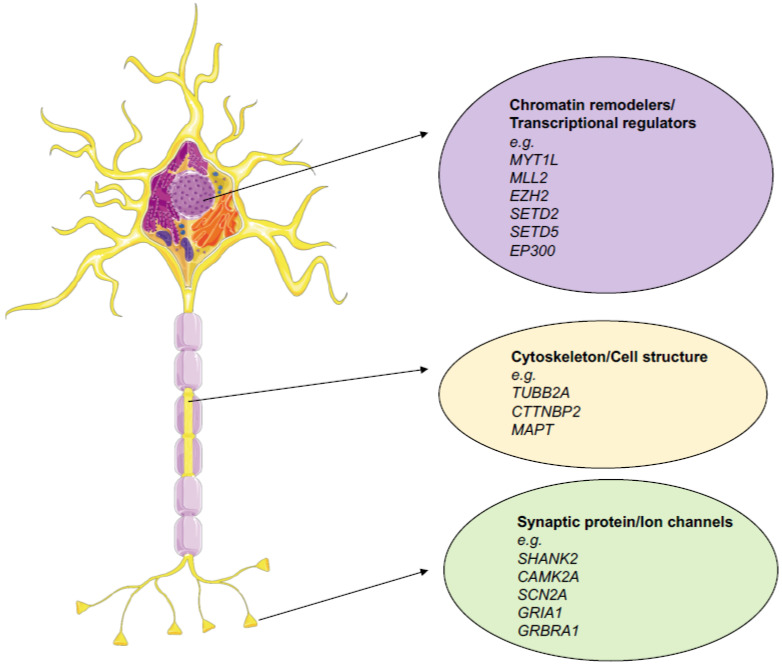
Main gene categories affected in NDDs with some relevant examples. Chromatin remodelers are genes that are implied to be involved in the regulation of chromatin in an activatory (*EP300, MLL2*) or repressive manner (*EZH2, MLL2*). Others control transcriptional processivity, such as (*SETD2* and *SETD5*). Cytoskeleton proteins such as *CTTNBP2* and *MAP2* are associated with the cytoskeleton and help to preserve its integrity. Others, such *TUBB2A,* are structural proteins that directly form its structure. Synaptic proteins and ion channels can be associated with different tasks, functionally active channels (*SCN2A*), neurotransmitter receptors (*GRIA1* and *GRBRA1*), calcium-responsive proteins (*CAMK2A*), and proteins that are associated with the synaptic structure (*SHANK2*).

Overall, three typical pathways have been identified to be the ones that are the most affected by mutations: the chromatin remodeling, transcription and splicing, cytoskeleton, and synaptic functionality pathways [16] (Figure 1). In this regard, a fascinating concept that has been introduced in this type of work are the gene networks that are associated with ASD pathogenesis. These are clusters show co-expression in tissue, indicating that they collaborate on a specific function together. For chromatin remodelers and transcriptional regulators especially, this concept is somehow pivotal because instead of having a direct role on brain development or function, they act more as orchestrators and regulators. Thus, understanding the context in which a gene is inserted and acts is essential if we are to understand the impact of function altering mutations. This expanded view could only be obtained through the use of high-level technology such as NGS.

Thanks to these large studies, associated genes were found to be related to either ASDs or IDs, despite many of the genes not being associated with ASDs or IDs before and many of whom were also poorly characterized from a functional standpoint. The massive amount of knowledge that has been gathered by these larger studies has granted the formulation of a new hypothesis on the molecular mechanisms operating in different pathologies.

It is worth mentioning that consequently, genes that were previously poorly characterized have gained interest within the scientific community. To list some examples, *SETD5* [18,19,20], *SHANK2* [21], *NCOR1* [22], and *MYT1L* [23] are examples of genes that have since become more frequently investigated using different in vitro and in vivo models in order to understand the normal biological and pathogenic mechanisms that are associated with them.

Thus, NGS has been pivotal not only for the discovery of new variants for diagnostic purposes but has also been pivotal in creating new biological questions.

### 2.3. NGS and Non-Coding Variant Interpretation

Despite the amount of information obtained by sequencing the coding portion of the genome, many NDD cases remain unexplained due to the failure to identify pathological mutations. Still, mutations in these patients may be present in the rest of the genome, they just may not be present in the area that is covered by WES. With the expansion of sequencing capacities to depict the whole genome, many significant variants within a non-coding genome have been linked to NDDs. It estimated that these variants now represent a huge chunk of NDD-associated mutations [24].

Although not directly coding for a specific gene, the non-coding portion of the genome has a very important role in regulating the where, how, and what is expressed in a type of tissue, such as the brain, at any given moment [25,26,27,28]. Indeed, around 98% of the genome is composed of non-coding elements, with more than 30% of it having a regulatory function [29]. This portion includes promoters, enhancers, miRNA binding sites, introns, and repeated sequences, among others. Several large-scale projects such as ENCODE [30] and roadmap epigenomics [31] have tried to classify these elements into different categories extensively. As consequence, it is now easier to infer the effect(s) of pathological mutations inside of those elements.

Genome-wide association studies (GWAS), which consist of whole genome sequencing (WGS), involve a variable number of patients and controls and are the most relevant type of investigations in this regard. Many of these have been conducted in the field of NDDs [32,33,34,35,36].

Considering that the heritable contribution in ASDs spans from 40% [37] to 80% [38,39], GWAS genetic studies have been instrumental in disentangling the etiology of these disorders. Common genetic variants [40], inherited gene disruptive mutations [41], and rare variants [32] are the main players in disease pathogenesis. Another smaller, but significant (around 10% to 30%) [17,41,42] contribution is represented by de novo mutations and copy number variations (CNVs). The latter corresponds to the regions of the genome that tend to vary in number between different individuals, which leads to structural variations being present between individuals in some cases. In some cases, these variations are associated with human pathologies [43].

Despite these extensive efforts, some issues remain present. Many studies, especially at the beginning, have been underpowered, the controls were not always accurately defined, and in some cases, the outcome was dependent on the statistical methods used to conduct the analysis. Another critical point refers to the interpretations of the biological meaning of these results. Despite the knowledge that is now available on the epigenome due to the above-cited ENCODE and roadmap studies, the interpretations that have been made, especially regarding the effects of single-nucleotide variants, remain challenging. Indeed, it is now known that some mutations can impair the binding capacity of a transcription factor (TF) or have a post-transcriptional effect through the alternation of the binding of the RNA-binding protein (RBPs). In other cases, the 3D topology of the chromatin, which is strongly believed to have a potential role in causing mendelian and non-mendelian diseases [35,44,45], may be affected.

An example of the effect of structural variants on the non-coding genome was described as being related to the *EOMES* gene (also known as *TBR2*), a marker of intermediate progenitors, the population of which is important during cortical development [46,47], as it has been found to be associated with familiar microcephaly [48]. In a recent work [25], the translocation (46,XY,t(3;10)(p24;q23)2x, 215kb upstream of *EOMES* TSS) associated with the disease was functionally characterized. More in detail, the enhancer sequences that directly control the expression of the *EOMES* gene are present within the region, and their translocation causes differences in chromatin bending and the consequent loss of contact between the promoter in its regulatory region. By deleting the same regions in human cells with CRISPR, the authors show how it strongly reduces the gene expression, clarifying the pathological process that is associated with this variant.

Another undervalued concept in understanding the mutation effect, especially for non-coding ones, is cell specificity. Indeed the consequences of mutations can vary depending on the cell type, as seen in Parkinson’s and Alzheimer’s disease [49]. Recently, this has also been proven in human cortical development [27]. Thanks to the use of a neural network approach (BPNet, [50]), the authors were able to describe the effect of mutations in a specific cell type using a large single-cell dataset coupled with the Simons Simplex Collection catalog, which comprises over 200,000 pathological variants. Starting from functional validation of non-coding sequences at the single cell level thanks to the transposase-accessible chromatin (ATAC-seq) assay, the algorithm used these data for the analysis, which helped in the prediction of which mutations might have a significant disruptive effect in a specific cell type and not in other cell types.

This work exemplifies the concept of how the data that have been produced previously can be integrated together with new technologies to obtain pieces of information that were not accessible before due to the technological limitations. The extensive use of machine learning is perfectly in line with this concept. To better understand the effects of non-coding mutations, a recently published study [51] used an approach enabling the interpretation of a previously described non-coding mutation effects, which was made available by SFARI, that allows the interpretation of similar variants for coding mutations. To do so, they put all of the data describing binding sequences of RNA and the DNA-binding protein into their model. In this way, the mutations could be classified as either disruptors of transcriptional regulation or as being able to alter the binding of the RNA-binding protein. As a result, it was possible to predict the functional effect associated with mutations that are otherwise more difficult to interpret.

Hereof, a significant contribution in the understanding of the effect and the origin of non-coding de novo somatic mutations has been recently provided by the group led Chris Walsh [52]. Through sequencing at ASD-affected human prefrontal cortex samples from a large cohort comprising 15 controls and 59 a very deep coverage (250×), the authors were able to study the somatic (also known as mosaic) mutations present in a small subset of cells. Interestingly, these mutations mark the progeny deriving from a single cell and was acquired in the early cell division stages post fertilization. Remarkably, 80 different somatic single-nucleotide variants are present in more than 2% of cells per individual on average. This led the authors infer that about half of the individuals that were tested might have one functional altering mutation inside a cortical region. Interestingly, individuals affected by ASDs present an excess of somatic mutations inside their neural enhancer sequences, indicating that the mosaic mutations inside the regulatory regions might have a role in disease pathogenesis.

Although these kinds of investigations are at a pioneer stage, they might contribute to elucidating the etiology of several NDD cases for which it is still not possible to produce a precise diagnosis. Importantly, considering that these mutations might only be present in a specific subset of cell populations in a specific organ, they may not have been detected in previous studies. This could be due to either lower sequencing coverage or to the use of other biological samples to obtain the sequenced DNA, such as blood or saliva.

This completely new layer of analysis exemplifies how almost 15 years after the technological evolution and deployment of NGS, we are probably still halfway, if not at the beginning, in our understanding of the information that we can extrapolate from our genome by using approaches based on it.

## 3. NGS in Experimental Modelling of NDDs

Besides the identification of the genetic causes of NDDs and their diagnosis, NGS has recently transformed basic research, especially research investigating disease models. The development of NGS-based technologies that are able to investigate not only genomic DNA but other source of materials such as RNA, miRNA, and DNA portion bound by specific proteins or methylated have been shown to flourish together with the establishment of different and versatile disease models.

**Figure 2:**
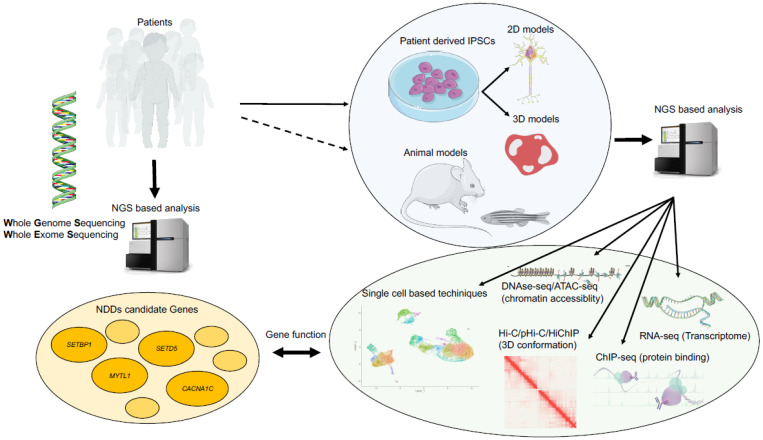
Investigation framework of NDDs fostered by NGS: from patient diagnose to basic pathogenic mechanism research.

Two big groups of models have come on to the scene in the last 15–20 years. The first part was dominated by animal (mainly mouse) models of several genetic diseases. Then, after the publication of the protocol to induce pluripotent stem cells (iPSCs) [53,54], a great deal of work has been conducted using patient cell-based models. This, of course, has also been possible thanks to great improvements in the development of effective differentiation protocols that are able to generate specific cell types as well as the 3D recapitulation of organs. The usage of an induced pluripotent stem cell (iPSC)-derived system their derived neural cultures in particular show great promise in the study of both the physiological mechanisms that govern normal neurodevelopment and the dysregulation of these mechanisms that may happen in NDDs directly in a human genetic background [55,56,57]. Intriguingly, the introduction of NGS technologies in the research field has greatly helped to improve iPSCs-based disease modelling by either (i) deepening the knowledge of gene networks, molecular pathways, and cellular signaling involved in physiological neurodevelopment that contain candidate genes that were previously associated with neuropsychiatric disorders and (ii) contributing to differentiation protocol development, allowing in vitro models to acquire features that are as similar to those found in the developing human brain as possible. The parallel development of modelling techniques on one side and analysis techniques such as NGS on the other side has reshaped research in the disease model area, as exemplified in Figure 2. In this chapter, we describe how they have been coupled together to generate new and important discoveries.

### 3.1. Transcriptomic Analysis

The possibility of analyzing the whole cell transcriptome at any moment with one single experiment is something that has changed how biomedical research is conducted. This is exactly what the advent of RNA-seq has brought to biomedical research. Specifically, this technique has been essential to characterize patient cell-derived models; this is partly because the timing of iPSC and NGS diffusion overlap and is also partly due to the effectiveness of the technique in capturing biological differences. The first published study using RNA-seq analysis to observed the transition between iPSCs to iPSC-derived neurons appeared in 2011 [58]; importantly, this transcriptomic profile revealed how cells in vitro undergo an extraordinary array of quantitative changes in gene expression during differentiation, similarly to what had already been reported in the human embryonic stem cell (hESC) system [59]. Interestingly, genes that are differentially expressed during this transition include transcription factors, chromatin remodelers, and players involved in cell adhesion and migration, are candidate risk factors for schizophrenia, bipolar disorders, and autism. This makes iPSC-based in vitro modelling an ideal tool for the study of the genetic alterations that occur in these pathologies. One of the novelties introduced by this key example study is that RNA-seq analysis provides many advantages over microarray, including the detection of low-copy transcripts, novel transcripts, long non-coding RNAs (lncRNAs), and splice isoforms [60,61]. For example, the authors discovered novel candidate lncRNAs that are involved in neurogenesis; some examples include HOTAIRM1 and HOTTIP, which were already known to be involved in regulating HOX gene expression, MALAT1, and RP11–319G6.1, which is transcribed in the opposite orientation from RBP1, the latter of which decreases in differentiating neurons. Thus, these novel candidate lncRNAs may also play a key role in neuropsychiatric disorders.

Later on, many studies took advantage of the combined use of whole transcriptomic and gene network analysis using the iPSC-based modelling of idiopathic and non-syndromic ASD [62,63], rare developmental syndromes [64,65], and other NDDs [66,67]. Here, both neural precursor cells and post-mitotic neurons show the dysregulation of expression in gene categories related to proliferation, intracellular signaling, extracellular matrix, synapsis, chromatin remodeling, differentiation and neurogenesis, and the control of oncogenes and onco-suppressors. Intriguingly, one of the first studies comparing the efficiency of in vitro neurogenesis with in vivo brain development in an unbiased manner used the RNA-sequencing technique to investigate the presence of differences between the two models. A highly conserved global gene expression and network architecture was reported. Preserved gene-modules were associated with ASD, supporting the utility of in vitro models for studying NDDs [68]. They also developed and validated a machine learning approach called CoNTExT, which can identify the developmental maturity and regional identity of the in vitro models to which different cellular systems and protocols could refer to in order to better interpret and explain in vitro results.

### 3.2. Epigenome Studies

Epigenomic investigation is another field of investigation in which the use of NGS technologies has grown quickly and has exploded in the last 5–10 years.

Some of these technologies exploit either the ability of an antibody to bind a specific protein or nucleic acid target, such as chromatin immunoprecipitation sequencing (ChIP-Seq) or methylated DNA immunoprecipitation sequencing (meDIP-Seq). Others handle some feature of the chromatin itself, such as accessibility, such as DNAse-seq [69], and more recently, the a transposase-accessible chromatin assay with high-throughput sequencing (ATAC-seq) [70]. Both techniques take advantage of the ability of enzymes to cut the DNA when it is free from protein binding. The first one is the oldest, and it is based in cutting the DNA using the DNAse enzyme that is only able to fragment the genome in the portions that are free from either the histones or the transcription factors, after which, the library is built and sequenced. The second is more recent and uses the same principle, but the enzyme in this case is the Tn5 transposase. The advantage offered by this technique is the lower input of cells required and the ability of the enzyme to not only cut the accessible portion of the chromatin but to also insert the adaptor sequences that are necessary for sequencing, thus making the library preparation process easier. These two techniques are essential when investigating the activity of the regulatory portion of the genome, especially when investigating the promoters and enhancers in that genome.

All of these techniques have been widely used to investigate several pathogenetic mechanisms that are associated with NDDs and development itself. Interestingly, as mentioned before, many NDD-associated disease genes function either as chromatin co-factors or transcriptional regulators, making it very enticing to use such approaches to investigate patient-derived cellular models. Additionally, investigating the epigenomic landscape adds a layer of information that, when coupled with other approaches, allows very complex mechanisms to be disentangled.

For example, the function of the *MYTL1*, a gene that has been associated with ASDs [16], was discovered thanks to a ChIP-seq based approach in an embryonic mouse brain and in fibroblasts reprogrammed into neurons [23]. This gene is a key regulator of neuronal differentiation; it exerts its function by binding and inhibiting the regulatory regions of non-neuronal genes, enabling differentiation toward the neuronal fate. ChIP-seq experiments together with RNA-seq have been the key to discovering this gene function along the genome. This work shows how combining different NGS-based approaches together facilitates our understanding of functions and mechanisms that were not previously characterized.

Another example related to chromatin regulation and transcriptional control is represented by the study of the *SETD5* gene. Studies in animal models carrying a *Setd5* haploinsufficiency have been performed to identify it as a fundamental epigenetic player that controls the transcriptional processivity in neural progenitors and neurons, illuminating the molecular events that connect epigenetic defects with neural dysfunctions at the basis of related human diseases [18,19]. For example, to understand how transcriptional processivity was affected, an adaptation of conventional RNA-seq was used, called DRB/Chromatin RNA-seq [19]. This technique exploits the pulse of a chemical substance (DRB) to block and then eventually release the RNA-polII activity. By extracting and analyzing the RNA produced from the treated cells at different time points, it is possible to measure how processivity is performed in different genotypes.

Another study that uses a combination of next-generation RNA sequencing, meDIP sequencing, ChIP-seq, and whole-genome miRNA analysis in human-derived fetal brain cells identified several levels of convergence between the molecular pathways affected by two different NDDs caused by the haploinsufficiency of *EHMT1* and *TCF4*, respectively [71]. The authors indeed identified mRNA and miRNA expression patterns that were more characteristic of differentiating cells than they were of proliferating cells, and the CpG clusters had similar methylation states in both reduced-gene dosage models, with a significant overlap in the gene targets of *TCF4* and *EHMT1* being observed. This highlighted the most salient features and provided avenues for similar treatments for NDDs caused by different genetic mutations.

As a final example, an interesting study combined chromatin accessibility investigation with a whole transcriptome. Here, the authors dissected the open regions dynamics in a human forebrain development model by applying ATAC-seq in combination with RNA-sequencing (RNA-seq) in order to map the epigenetic and gene expression signatures of the neuronal and glial cell lineages [26]. Indeed, gene expression changes and its control by accessible chromatin during human brain development is of great interest because it is also known that the disruption of these cellular events by either genetic or environmental factors can lead to neurodevelopmental disease, including ASDs and IDs. However, a lack of available primary brain tissue samples, especially those taken at later stages of the disease, as well as the limitations of conventional in vitro cellular models have precluded a detailed mechanistic understanding of corticogenesis in healthy and disease states. Through the use of three-dimensional the directed differentiation of hiPSCs into the dorsal and ventral forebrain organoids as well as primary brain tissue samples, the authors could reveal the chromatin state transitions in vitro that are closely related to human forebrain development in vivo, which was previously unappreciated. They subsequently used this resource to map the genes and genetic variants that are associated with schizophrenia and autism spectrum disorders to distinct accessibility patterns to reveal cell types and periods of susceptibility. They were able to reveal concomitant progressions in motif accessibility over time during the cortical development of different transcription factors: the EOMES, TBR1, and NEUROD2 motifs represented the earliest phase of corticogenesis, TCF4 and MEF2C represented a middle phase, while the FOXP1, CUX2, and POU3F2 (BRN2) motifs represented the latest stages. For example, *MEF2C* has been previously associated with ASD [72], but its gene regulatory activity in the context of human cortical development has not been described previously. They indeed found that *MEF2C* was not expressed in progenitor cells or CTIP2+ neurons at 75 days in dorsal cortical organoids (early phase); however, it partially colocalized with CTIP2 and RORB after 130 days of differentiation (middle phase). These convergent lines of evidence suggest an important role for *MEF2C* in midcortical neurogenesis.

### 3.3. D Chromatin Structure Investigation

Three-dimensional physical interactions of discrete regions within chromosomes dynamically regulate gene expression in a cell-specific manner [73,74,75]. Even single point mutations that may disrupt or impact chromatin organization could likely contribute to complicating the genetic landscape of NDDs. Indeed, based on the changes of the 3D chromatin structure, the putative associated genes for several diseases can be identified, and can thus help to formulate hypotheses on pathological mechanisms [76,77]. A fundamental NGS-based technique has been at the forefront of these discoveries; this technique is called high-throughput chromosome conformation capture (Hi-C) [75]. Similar to ChIP-seq, it is based on fixed cells whose genome is digested using a restriction enzyme; the obtained fragments are then repaired using biotinylated nucleotides, are purified, and are then sequenced at a very high coverage. This technique has been successfully applied, for example, to monitor the changes in the 3D chromatin-structure that can take place during the in vitro process that differentiates iPSCs to NPCs both in mice and humans. The presence of differentially interacting regions in both cell types has been found, thus suggesting that spatial organization affects the gene regulation of both pluripotency maintenance and neuroectodermal differentiation as well as the transition from precursors to terminally differentiated neurons [78,79]. Chromosomal conformation changes during in vitro differentiation from NPCs to neurons or astrocytes revealed that the majority of these conformational plasticity changes (i) occur in genes that regulate neuronal connectivity and chromatin remodeling, (ii) are cell specific, and (iii) include interactions that are anchored in common variant sequences that confer a heritable risk for schizophrenia in neurons [80].

Due to the complexity of the analysis and the requirement for a massive sequencing depth, several HiC-based alternatives have been developed to try to address 3D conformation in a more affordable manner. An interesting variation is the HiChIP [81], which fuses ChIP-seq and Hi-C together. In this case, before performing the precipitation of biotinylated DNA fragment, the immunoprecipitation of a protein of interest is performed, thus only isolating the 3D interactions that are associated with a specific protein. The advantage consists of a reduced sequencing depth and a less complicated dataset.

Another approach that focuses specifically on interaction involving regulatory elements is the promoter capture Hi-C (pHiC) technique. In this case, before purifying the ligated fragments, RNA oligonucleotides are used to isolate specific promoter sequences; thus, the interactions only involve them and their regulatory regions. PHiC has been used to investigate the enhancer–promoter dynamics during the differentiation of several neural lineages arising from IPSCs [82]. The work was performed by investigating chromatin interactions, open chromatin regions, and transcriptomes using pHiC, ATAC-seq, and RNA-seq, respectively, in four functionally distinct neural cell types: iPSC-induced excitatory neurons, lower motor neurons, iPSC-derived hippocampal dentate gyrus-like neurons, and primary astrocytes. The authors were thus able to identify the promoter*–*enhancer pairs in different neuronal and astrocytic subtypes and to understand the regulatory dynamics that take place during this process and their functional effect. These regulatory interactions were further confirmed by overlapping them with VISTA [83], a database containing experimentally validated enhancer sequences. Finally, to validate their findings, the CRISPRi approach was used to epigenetically inactivate those regions, thus altering the regulator network to confirm their importance for the biological processes that were investigated.

### 3.4. Single-Cell Approaches

The technological revolution that is currently changing life science, including research involving neural development and NDDs, is single-cell analysis. This innovation would have not been possible without NGS technologies.

Starting from the first published mRNA-seq whole-transcriptome analysis of a single cell in 2009 [84], evolution has been fast, and nowadays, thanks to different custom and commercially available platforms, single-cell analysis has expanded to epigenomics and whole-genome interrogation.

This technique has been widely used in order to increase the reliability of experimental models, such as iPSC-derived systems, to represent the features of the developing human brain and to study their associated diseases [85,86,87]. For example, scRNAseq revealed successful differentiation protocols to obtain in vitro neuronal cultures that were mature enough to sustain AMPAR- and NMDAR-mediated synaptic transmission [88], which is useful to model glutamate-receptor related-dysfunctions (such as schizophrenia, epilepsy and ASD). Transcriptome profiling at single cell levels have been also employed to dissect the transcriptomic signatures of different time-points of in vitro neural differentiation (e.g., self-renewal, early neuronal differentiation, neural precursor cells, assembled rosettes, and differentiated neuronal cells), comparing them with human brain tissue [89] and revealing the ability of this system to faithfully model human neurogenesis and neuronal maturation.

However, the power of single-cell approaches is well exploited in the study of the tri-dimensional (3D) context, both of native organs and 3D neural cultures [90,91]. Indeed, although the 2D human-specific cultures were and are widely used and helped gain knowledge of human brain development, they have limitations in recapitulating the complexity of the structure and the cellular diversity of the brain, thus losing information regarding spatial architecture, cell to cell interactions, and neuronal connectivity. In order to overcome these drawbacks, the progress with human iPSC-derived cellular models has stimulated the generation of more complex cultures, which are often referred to as organoids, with the aim of gaining a 3D structure with a spatial and cytoarchitecture that more closely resembles human organs. These cultures take advantage of the self-organization process that guides organogenesis. Since the first trials more than 10 years ago [92,93], several variations have been proposed to optimize the original organoid protocol. Nowadays, two main protocols exist, with organoids that can be generated either by following undirected differentiation methods (“intrinsic protocols”) that omit inductive signals and generate brain regions driven by intrinsic mechanisms [94,95,96,97] or by patterning through directed differentiation approaches (“extrinsic protocols”) that guide the formation of region-specific brain organoids or spheroids [98,99,100,101]. Independently from the chosen protocol, organoids have so far been extremely useful for investigating early brain development in vitro better, and by implication, the study and understanding of disorders that are related to the early stages of neurodevelopment. As for classical models, organoids have been used extensively to study neurodevelopmental and neuropsychiatric disorders, including microcephaly [94,102], macrocephaly [103], lissencephaly [103,104,105], periventricular heterotopia [106,107,108], ASD [109], schizophrenia [110], Rett syndrome [111], fragile X syndrome [112], frontotemporal dementia [113], Schinzel–Giedion syndrome [65], and many others.

Nowadays, the forefront application of single cell-omics is the integration between them, both at the analytical and experimental levels. The combination of different techniques at the single-cell resolution (e.g., single-cell RNA-seq and single nucleus ATAC-seq) can be employed to validate organoids as models to study neurodevelopment, NDDs, and brain evolution. The group led by Prof. Paola Arlotta performed single-cell RNA-seq from several individual organoids (accounting for more than 160,000 cells) and found that 95% of the organoids generate a virtually indistinguishable compendium of cell types, following similar developmental trajectories and with a degree of organoid-to-organoid variability that is comparable to that of individual endogenous brains [114]. Then, this combination of single cell methods was also useful in mapping the epigenetic and gene expression signatures of neuronal and glial cell lineages over 20 months of in vitro differentiation. The resulting data were correlated with genetic variants that were associated with schizophrenia and ASD, revealing affected cell types and periods of susceptibility [26]. Similar methods were been previously used on cortical tissue derived from patients with autism to identify autism-associated transcriptomic changes in specific cell types [115], obtaining comparable results that further increase the reliability of the experimental modelling of NDDs through organoids. Furthermore, cerebral organoids (obtained both from human and different primates) were also employed in comparative evolution studies, in which single-cell transcriptomics and accessible chromatin profiling helped to investigate either primates or human-specific gene-regulatory changes. These contribute to the compilation of a temporal cell atlas of forebrain development, allowing us to take a step forward in understanding the dynamic gene-regulatory features that are unique to human neurodevelopment and that are possibly affected in NDDs [116].

The integration of multiple single cell approaches has been recently used to reveal new insights into the human cortical development by directly applying it to primary human embryonic brain tissue [27]. Here, a huge ATLAS of single cells transcriptomes (57,868) and epigenomes (31,304) spanning four different gestational times were generated. This study helped to elucidate how key TFs expression and function are a potent determinant of the cell differentiation trajectory. Something that was particularly interesting was that the connection that was described between the expression and activity of these TFs and other genes, which was validated experimentally using the multiome approach that allows the transcriptome and chromatin accessibility deriving from the same single cell to be profiled at the same time. This paradigm has been applied to describe the developmental trajectory of cortical neurons, describing different waves of transcription factors during this differentiation process. This analysis comprised of evaluating the changes taking place in the open chromatin during the process at the same time. This approach also more broadly helped to connect different gene expression modules and chromatin accessibility that defines commitment toward different cell populations that are present in the cerebral cortex. Something that is particularly interesting is the description of two transcriptional modules associated with two different subtypes of astrocyte precursors that were not associated with specific areas but that were possibly associated with a different specification program in the adult brain.

These data were recently implemented using a similar experimental framework in another work [28] focusing on different brain areas. By combining data regarding chromatin accessibility and transcriptomics at the single-cell resolution, the genomic loci that direct the development brain cells originating from different areas were discovered and characterized. Furthermore, the huge ATLAS of single cells that were generated was used as a benchmark to understand the fidelity of organoids in recapitulating the trajectory of the brain development of different cell types.

The development in single-cell sequencing techniques have allowed the most detailed consensus ATAS of the mouse motor cortex to date to be produced. These results were able to be achieved thanks to the BRAIN Initiative Cell Census Network (BICCN) [117]. This ATLAS contains the transcriptome, chromatin accessibility, DNA methylation, and spatially resolved transcriptome integrated together at the single-cell level and contains information regarding cell connectivity and electrophysiology. This kind of initiative represents how the next step is represented by the integration of a different data source, which requires a single-cell resolution to have a complete view of different aspects regarding the structure and function of a specific organ, in this case, the brain.

Technological advancements are often the key for groundbreaking achievements in biological research. Examples of this are the ameliorations in microscopy and genetic engineering. We believe that with the NGS technique, we are at the forefront of one of such technical revolution permitting the discovery of new pathological variants, incredibly complex molecular phenotypes, and even the design and use complex screening methods (e.g., using CRISPR/Cas9) [118,119,120].

Specifically, spatial transcriptomics offer a powerful tool that is only it is infancy and that is already helping to solve one of the most evident problems related to normal single-cell, analysis which is establishing the origin of the cells analyzed, and this new technology will definitely improve our ability to understand the function of complex tissue even more [121].

## 4. Conclusions

In this review, we have summarized some of the beneficial effects resulting from the use of NGS in the diagnosis and in basic research of NDDs. Please note that is almost impossible to be exhaustive in this regard since either a technique or a new adaptation is implemented virtually every day. For example, a new line of usage of NSG is spatial transcriptomics which was nominated as method of the year 2020 by *Nature Methods*. This evolution of single-cell genomics couples the ability to discriminate single cells and the possibility acquiring the information of where those same cells are located within a tissue. In the words of the Allen Institute for Brain Science director Hongkui Zeng and researcher Bosiljka Tasic, working with single-cell genomics instead of bulk techniques is more similar to facing a fruit salad than a smoothie, while spatial transcriptomic is a fruit tart. The continuous technical improvements and the smart applications from scientists will soon lead to other spectacular achievements that will perhaps allow it to be possible to follow the tart during its preparation, life, and consumption.
